# Bloom Syndrome Protein Activates AKT and PRAS40 in Prostate Cancer Cells

**DOI:** 10.1155/2019/3685817

**Published:** 2019-05-09

**Authors:** Kun Chen, Houqiang Xu, Jiafu Zhao

**Affiliations:** ^1^Key Laboratory of Animal Genetics Breeding and Production in the Plateau Mountains Region, Ministry of Education, Guizhou University, Guiyang 550025, China; ^2^Center Lab, Guizhou Provincial People's Hospital, Guiyang 550003, China; ^3^College of Life Science, Guizhou University, Guiyang 550025, China

## Abstract

**Purpose:**

Prostate cancer (PC) is a common malignant tumor and a leading cause of cancer-related death in men worldwide. In order to design new therapeutic interventions for PC, an understanding of the molecular events underlying PC tumorigenesis is required. Bloom syndrome protein (BLM) is a RecQ-like helicase, which helps maintain genetic stability. BLM dysfunction has been implicated in tumor development, most recently during PC tumorigenesis. However, the molecular basis for BLM-induced PC progression remains poorly characterized. In this study, we investigated whether BLM modulates the phosphorylation of an array of prooncogenic signaling pathways to promote PC progression.

**Methods:**

We analyzed differentially expressed proteins (DEPs) using iTRAQ technology. Site-directed knockout of BLM in PC-3 prostate cancer cells was performed using CRISPR/Cas9-mediated homologous recombination gene editing to confirm the effects of BLM on DEPs. PathScan® Antibody Array Kits were used to analyze the phosphorylation of nodal proteins in PC tissue. Immunohistochemistry and automated western blot (WES) analyses were used to validate these findings.

**Results:**

We found that silencing BLM in PC-3 cells significantly reduced their proliferative capacity. In addition, BLM downregulation significantly reduced levels of phosphorylated protein kinase B (AKT (Ser473)) and proline-rich AKT substrate of 40 kDa (PRAS40 (Thr246)), and this was accompanied by enhanced ROS (reactive oxygen species) levels. In addition, we found that AKT and PRAS40 inhibition reduced BLM, increased ROS levels, and induced PC cell apoptosis.

**Conclusions:**

We demonstrated that BLM activates AKT and PRAS40 to promote PC cell proliferation and survival. We further propose that ROS act in concert with BLM to facilitate PC oncogenesis, potentially via further enhancing AKT signaling and downregulating PTEN expression. Importantly, inhibiting the BLM-AKT-PRAS40 axis induced PC cell apoptosis. Thus, we highlight new avenues for novel anti-PC treatments.

## 1. Introduction

Prostate cancer (PC) is a common malignancy of prostate epithelial cells [1]. PC is the most common cancer affecting American males, with 221,000 newly diagnosed cases and 27,500 deaths reported in 2015 alone [2]. In China, the rising average age of the population in combination with lifestyle changes have contributed to a clear upward trend in PC incidence and mortality [3]. PC is highly hereditary, and genetic PC risk factors can be passed from parents to their children [4]. PC is also a complex disease, and these genetic variants interact with environmental factors and dietary habits [5]. Active surveillance, radical prostatectomy, and radiation therapy are common treatment choices for localized PC. Chemotherapy drugs which target signaling pathways with a known association to PC tumor progression, including mTOR, PI3K-Akt, MAPK, AMPK, and p53 signaling, are used to induce PC cancer cell death. This is exemplified by BEZ235, a phosphatidylinositol-3-kinase (PI3K)/mammalian target of rapamycin (mTOR) inhibitor that blocks AKT phosphorylation (Thr308/Ser473) and can prevent breast [6, 7], glioma [8], and non-small-cell lung cancer growth [9, 10]. Combining BEZ235 with abiraterone acetate, which blocks cytochrome P450 17 alpha-hydroxylase to significantly reduce androgen production, improves therapeutic outcomes in PC [11]. However, PC therapy remains ineffective overall, and more effective alternative treatments are urgently required [12].

DNA helicases within the RecQ protein family are involved in genome maintenance. These proteins, which are highly conserved from bacteria to humans, aid in maintaining genetic stability [13, 14]. RecQ helicases in human cells include RECQ1, BLM, WRN, RECQ4, and RECQ5. Defects in the WRN helicase are linked to a form of progeria associated with accelerated aging phenotypes termed Werner syndrome (WS). In contrast, mutations in Bloom syndrome protein (BLM) can result in the autosomal recessive disease Bloom syndrome (BS) [15, 16]. Unlike WS patients, BS patients do not exhibit a progeria phenotype but instead are prone to develop multiple malignancies including breast, prostate, and lung cancers [17, 18]. RecQ helicases are highly expressed in tumor cells, and silencing of the WRN helicase by RNA interference (RNAi) facilitates the treatment of many cancer types [19, 20]. Studies have also shown that BLM is highly expressed in breast cancer tissue and represents a novel breast cancer biomarker [21, 22]. Nonsense mutations in the BLM gene increase the risk of PC [23], and BLM expression is associated with PC susceptibility in the Chinese population [24]. Previous work has shown that knockdown of BLM inhibits PC cell proliferation and promotes PC apoptosis. However, the mechanism by which BLM may contribute PC tumorigenesis remains undetermined.

Given the close relationship between the WRN and BLM helicases, and the fact that the silencing of both is used in the treatment of multiple forms of cancer, it stands to reason that certain shared pathways govern the link between these two proteins and cancer cell proliferation. Oxidative stress has been proposed as a fundamental driver both of aging and cancer [25, 26]; thus, the shared oxidative stress-response pathway is a likely common link given that reactive oxygen species- (ROS-) mediated damage can substantially disrupt cellular protein function and DNA integrity.

The aim of the present study was to dissect the role of BLM in PC progression. To achieve this, iTRAQ technology was used to examine differentially expressed proteins (DEPs) among PC, normal prostate, and benign prostatic hyperplasia tissue samples. We confirmed that BLM was significantly overexpressed in PC cells and that it regulated the phosphorylation of an array of important cellular kinases. BLM was then silenced using CRISPR/Cas9 [27–30] in PC-3 prostate cancer cells to observe its effects on PC cell health, proliferation, and signaling. We found that BLM deletion inhibits PC cell proliferation via downregulating pAKT (Ser473) and pRAS40 (Thr246), which was accompanied by enhanced ROS production. We further show that AKT and PRAS40 inhibition also inhibit BLM and increase ROS levels, indicating that these signaling pathways are regulated by BLM through a positive feedback loop. These results give novel molecular insights into the role of BLM during PC tumorigenesis and lay the foundation for targeting the BLM-AKT-PSRAS40 axis during PC treatment. They also highlight avenues for future research, identifying ROS mechanisms acting in concert with BLM to facilitate PC oncogenesis, as key potential targets for future combinatorial therapy approaches.

## 2. Materials and Methods

### 2.1. Tissue Specimens

A total of 43 patients were included in this study. Tissue specimens were collected at the Department of Urology of Guizhou Provincial People's Hospital with informed consent from the patients and approval from the ethics committee. All procedures were performed in accordance with the Declaration of Helsinki principles and relevant policies in China. In total, 10 patients in the PC group underwent radical prostatectomy. The prostatic lesion was collected and confirmed as PC by pathological diagnosis. 15 patients underwent laparoscopic radical cystectomy. The prostatic tissue was collected, and the absence of cancer cell infiltration was confirmed by laboratory analysis. 18 patients with benign prostatic hyperplasia were treated via transurethral resection of the prostate and were confirmed with benign prostatic hyperplasia by laboratory analysis.

### 2.2. Cell Lines and Culture Conditions

The human prostate cancer cell lines PC-3, VCAP, and LnCAP and the human prostatic hyperplasia cell line BPH-1 were purchased from ATCC. LnCAP cells were cultured in RPMI 1640 medium supplemented with 10% fetal bovine serum (FBS), 100 U/mL penicillin, and 100 mg/mL streptomycin. All other cell lines were cultured in Dulbecco's Modified Eagle's Medium (DMEM) supplemented with 10% FBS, 100 U/mL penicillin, 100 mg/mL streptomycin, and 2 mM glutamine. All cell lines were cultured at 37°C and 5% CO_2_.

### 2.3. CRISPR/Cas9-Mediated Gene Editing

CRISPR/CAS9 vector backbones, donor vector backbones, and T7E1 primers were synthesized by Beijing CasGene Biotech Co. Ltd. T4 ligase was purchased from Thermo Fisher Scientific. T7 endonuclease I was purchased from Beijing ViewSolid Biotech Co. Ltd. Polymerase chain reaction (PCR) systems were purchased from Becton Dickinson Inc. We analyzed a panel of 5 sgRNAs and selected the most efficient sgRNA (sgRNA3: TCACTTGATGGCCCTATGGA). This was used to construct the expression vector via the following primer sequences: (5′-3′) F: GTGGGAACGAACTGCTTCAG and R: TCTTGGTGTTTCAGCCCAGT. The donor vector was constructed by designing homology arms based on the sequences upstream and downstream of the sgRNA3 nick (Supplementary Figures 1–5).

### 2.4. iTRAQ

Samples were prepared for iTRAQ experiments using the SDT lysis method. SDT lysis buffer was added to samples followed by 15 mins of sonication in a boiling water bath. Samples were centrifuged at 14,000*g* for 15 mins, and protein concentration in the supernatant was quantified via bicinchoninic acid (BCA) assay. Samples were stored at -80°C until use. Samples were further digested by filter-aided sample preparation (FASP), and protein concentrations were assessed in each fraction. For each sample, 100 *μ*g of total peptide was labeled using the AB SCIEX iTRAQ Labeling Kit. The normal prostate group was labeled with tags 113 and 114; the benign prostatic hyperplasia group was labeled with tags 115, 116, and 117; and the PC group was labeled with tags 118, 119, and 121. Labeled peptides from each group were mixed and fractionated using an Agilent 1260 infinity II HPLC system, separated using an nanoliter flow rate Easy-nLC system, and analyzed using a Q Exactive Plus mass spectrometer. Identification and quantitative analysis were performed using the Mascot 2.5 and Proteome Discoverer 2.1 software. Differentially expressed proteins (DEPs) underwent Gene Ontology (GO) functional annotation (database version: go_201504.obo) and KEGG pathway annotation. The distributions of each GO class or KEGG pathway for these DEPs and for overall protein sets were compared by Fisher's exact tests to evaluate the significance of enrichment. Proteins which were differentially expressed among prostate cancer, normal prostate, and benign prostatic hyperplasia tissue samples were identified using the iTRAQ technology (Supplementary Table 1).

### 2.5. BLM-shRNA Vectors

We constructed a short hairpin RNA (shRNA) molecule targeting the Bloom helicase using the mammalian expression plasmid vector CMV-copGFP-T2A-Puro-H1-mcs. Successful construction of the vectors was validated by DNA sequencing prior to synthesis of the RNAi vectors by the Shanghai Gemma Medical Technology Dev. Co. Ltd. Sequences were as follows: shRNA-1: 5′-GCA GCG ATG TGA TTT GCA TCG TTC AAG AGA CGA TGC AAA TCA CAT CGC TGC TTT TTT G-3; shRNA-2: 5′-GCT TCA GCA GCG GAA CAT AAG TTC AAG AGA CTT ATG TTC CGC TGC TGA AGC TTT TTT G-3′. Both of these shRNAs silenced BLM expression, with respective efficiencies of 33% and 51% as confirmed in our previous studies (Supplementary Figure 5).

### 2.6. Antibody Array Detection

Arrays were performed using the PathScan® Antibody Array Kit (Cell Signaling Technology, 7323) according to the manufacturer's instructions. Loading concentrations were adjusted to 0.2-1.0 mg/mL. All array experiments were conducted in triplicate.

### 2.7. Reagents

The PRAS40 inhibitor GDC0068 (mw: 530.91, mf: C_24_H_34_CL_3_N_5_O_2_), the AKT inhibitor BEZ235 (mw: 469.53, mf: C_30_H_23_N_5_O), and the chemotherapeutic drug cisplatin (CDDP) (mw: 300.05, mf: CL_2_H_6_N_2_Pt) were purchased from MedChemExpress.

### 2.8. Apoptosis Detection and CCK-8 Drug Sensitivity Assessment

Cells were seeded into 96-well plates at a density of 5,000 cells per well for CCK-8 assays. Adherent cells were treated with various concentrations of BEZ235, GDC-0068, and CDDP for 48 h in culture media. Next, the CCK-8 reagent was added to each well for 2 h, after which the absorbance at 450 nm was measured using a microplate reader.

Annexin V-FITC/PI staining was used to assess cellular apoptosis. Briefly, cells were seeded into 6-well plates at a density of 20,000 cells per well. Adherent cells were treated with BEZ235, GDC-0068, and CDDP in the culture media for 24 hrs. Then, staining was performed using an Annexin V-FITC/PI Cell Apoptosis Assay Kit according to the manufacturer's instructions. Stained cells were detected on a flow cytometer (CytoFLEX).

### 2.9. Intracellular ROS Detection

The nonfluorescent probe DCFH-DA was used to monitor ROS production. DCFH-DA diffuses into cells where it is deacetylated by esterases to form the nonfluorescent product DCFH. In the presence of ROS, DCFH reacts with ROS to form DCF, a fluorescent product, and the resulting fluorescence is proportional to ROS levels. For the measurement of intracellular ROS, cells were treated with H2DCF-DA (10 *μ*M) for 30 min at 37°C in the dark, and washed twice with cold PBS. Intracellular ROS production was then analyzed using flow cytometry (excitation, 485 nm; emission, 530 nm).

### 2.10. Immunohistochemistry and Automated Western Blot Analysis

Anti-pAKT (Ser473, 60 kDa, isotype: rabbit), anti-pPRAS40 (Thr246, 40 kDa, source: rabbit), anti-AKT (60 kDa, isotype: rabbit), and anti-PRAS40 (40 kDa, source: rabbit) antibodies were purchased from Cell Signaling Technology. Anti-BLM antibodies (159 kDa, source: rabbit) were purchased from Abcam. Anti-*β*-actin antibodies (49 kDa) were purchased from Santa Cruz Biotechnology Inc. Antibodies were used at the following dilutions: BLM, 1 : 500; P-AKT, 1 : 50; and P-PRAS40, 1 : 200. Staining intensity criteria were as follows: 0 (-), 1 (weak), 2 (moderate), and 3 (strong). The positive staining rates were scored as follows: 0 (negative), 1 (1-25%), 2 (26%-50%), 3 (51-75%), and 4 (76%-100%). An automated western blot quantitative analyzer (ProteinSimple) was used to assess protein expression according to standard protocols (antibody dilution factor: 1 : 50). A grayscale analysis of the band intensities was then performed using Compass software.

### 2.11. Statistical Analysis

The distributions of each GO class or KEGG pathway among the DEPs and total proteins were compared using Fisher's exact test to assess the enrichment significance. One-sided or two-sided paired Student's *t*-tests were performed for single comparisons. All quantitative results were expressed as the mean ± SD. A *P* value < 0.05 was considered statistically significant.

## 3. Results

### 3.1. iTRAQ-Mediated Analysis of DEPs in PC Samples

**Figure 1:**
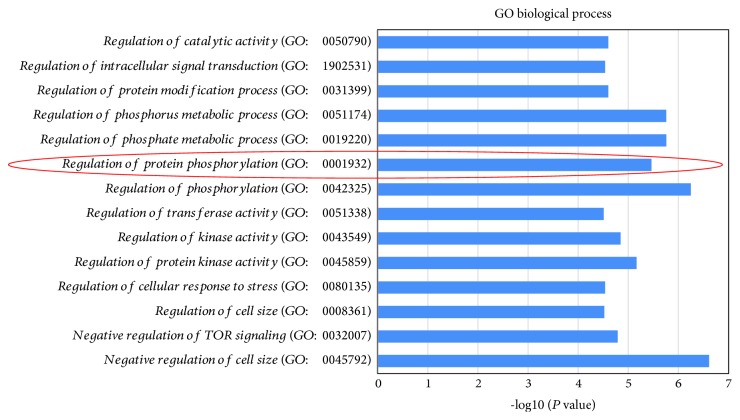
Enrichment of the DEPs in biological processes. The regulation of protein phosphorylation (GO: 0001932) was significantly enriched, indicating its involvement in the development and progression of PC (*P* < 0.05).

iTRAQ technology was used to uncover DEPs among prostate cancer, normal prostate, and benign prostatic hyperplasia tissues [31, 32]. A total of 21 DEPs (fold change >1.2 or <0.8, *P* value < 0.05) for which protein expression was up- or downregulated in cancerous tissue were identified (of which 17 proteins were upregulated and 4 were downregulated). Functional annotation was performed for these DEPs using the GO database. Whether DEPs were significantly enriched among specific biological processes (BPs) was analyzed using Fisher's exact test (Figure 1), revealing that protein phosphorylation (GO: 0001932) was significantly enriched. An analysis of the DEPs involved in the regulation of protein phosphorylation revealed high BLM expression in PC cancer tissue (Table 1). We also performed KEGG pathway annotation of these 21 DEPs and screened the target pathways following enrichment analysis (*P* < 0.05, ≥4 target proteins involved in the pathway) (Table 2). Among these, known oncogenic pathways including the mTOR [33], PI3K-Akt [34], MAPK, AMPK, [35], and p53 pathways were identified, indicating potential links to ROS production as related to tumor growth and alterations in cellular translational activity worthy of future investigation. In addition, all of these pathways have well-characterized links to cancer cell proliferation, differentiation, apoptosis, and DNA damage repair [36].

### 3.2. Site-Directed Knockout of BLM Inhibits PC-3 Cell Proliferation

**Figure 2:**
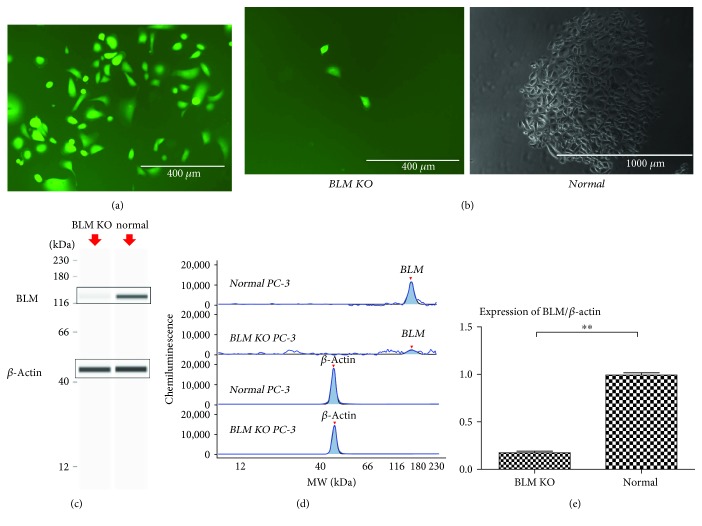
Site-directed knockout of the BLM gene in PC-3 cells. (a) PC-3 cells were observed under fluorescence microscopy following transfection with BLM CRISPR/Cas9 expression and donor vectors. (b) Monoclonal PC-3 cell clusters are shown after 3 weeks of culture. Normal PC-3 cells undergo monoclonal proliferation, while BLM KO PC-3 cells do not. (c) Expression of BLM in different PC-3 cell lines. Results were generated using the WES system. Band intensities represent protein expression. (d) Chemiluminescence was used to generate the peak area and molecular weight data by linear analysis using the WES system. (e) The BLM/*β*-actin ratio was significantly decreased in BLM KO PC-3 cells (^∗^ *P* < 0.01 and ^∗∗^ *P* < 0.01).

To examine the role of BLM in PC progression, we generated a BLM knockout human PC-3 cell line using CRISPR/Cas9 [37]. We initially designed five Cas9/sgRNA expression vectors, and the activity of each was detected using T7 endonuclease I. Based on these screening assays, we determined that sgRNA3 was the most active. Next, donor vectors were designed based on the sequences upstream and downstream of sgRNA3. The expression vectors and donor vectors were cotransfected into PC-3 cells, and these cells underwent ampicillin selection (6 *μ*g/mL for 6 days) 48 h posttransfection, followed by the selection for the donor vector using puromycin (0.6 *μ*g/mL for 5 days). The donor vector expressed enhanced yellow fluorescent protein (eYFP) driven under control of the hEF-1*α* promoter, and as such, remaining cells were screened for green fluorescence (Figure 2(a)) confirming successful vector integration into the PC-3 cell genome in BLM KO cells.

Normal PC-3 cells can undergo monoclonal proliferation, and 3 weeks of cell growth results in the formation of readily visible monoclonal cell clusters. To rule out any off-target effects due to plasmid transfection, we cotransfected PC-3 cells with the plasmid backbone and selected for resistant cells which did not undergo gene recombination. These control cells formed a monoclonal cell mass after 3 weeks, indicating that plasmid transfection alone had no effect on the ability of PC-3 cells for monoclonal proliferation. However, monoclonal screening of the BLM KO PC-3 cells revealed a reduction in proliferative capacity. The cells proliferated slowly, aged, became vacuolated, and died (Figure 2(b)). Because these KO cells could not form monoclonal clusters, sufficient numbers of BLM KO PC-3 cells could only be obtained via repeated transfections. After collecting a sufficient number of BLM KO PC-3 cells, changes in BLM helicase expression between BLM KO and wild-type PC-3 cells were assayed using the automated western blot analysis system (WES) [38, 39]. We observed significantly lower BLM expression in the BLM KO PC-3 cells as compared to wild-type cells (*P* < 0.01) (Figures 2(c)–2(e)). Taken together, these data confirmed that knockout of BLM was successful and it impaired the proliferation and viability of PC-3 cells.

### 3.3. Screening for Significant Differences in Phosphorylated Proteins

**Figure 3:**
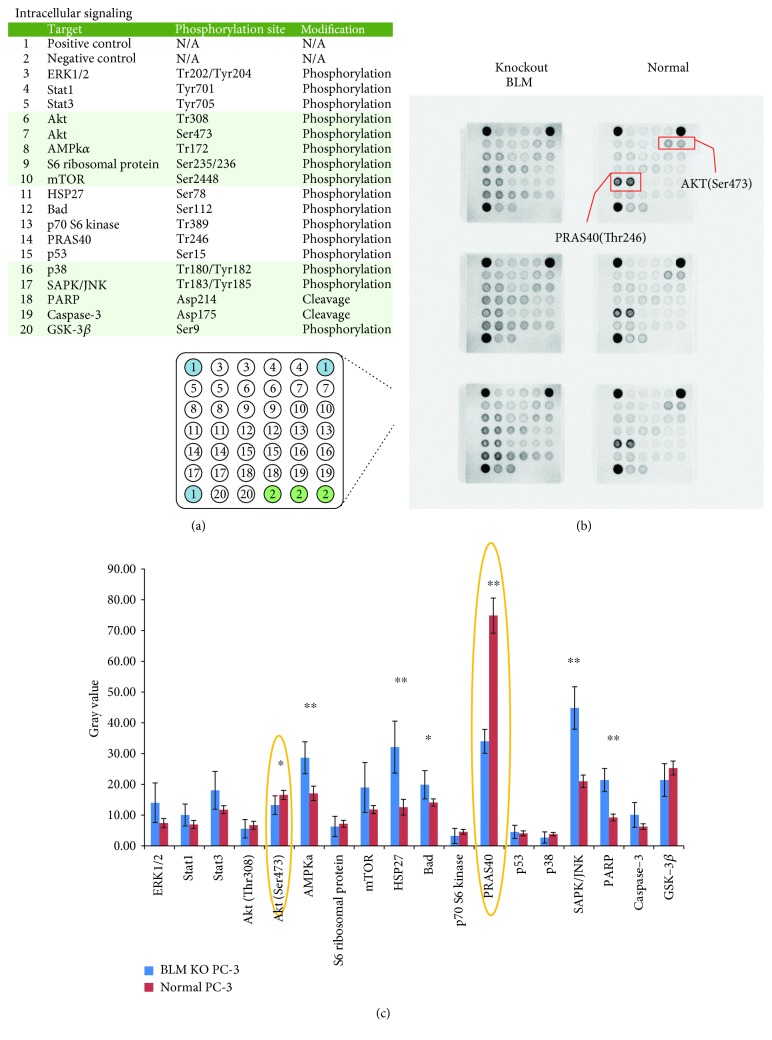
Selected phosphorylated nodal proteins in PC-3 and BLM KO PC-3 cells. (a) Phosphorylated nodal proteins investigated in this study. (b) Expression of phosphorylated signaling proteins in two different PC-3 cell lines. (c) Quantitation of AMPK*α*, HSP27, Bad, SAPK/JNK, and PARP expression levels revealed their upregulation in BLM KO PC-3 cells, while AKT (Ser473) and PRAS40 (Thr246) expression levels were significantly downregulated (mean ± SD; ^∗^ *P* < 0.05 and ^∗∗^ *P* < 0.01).

**Figure 4:**
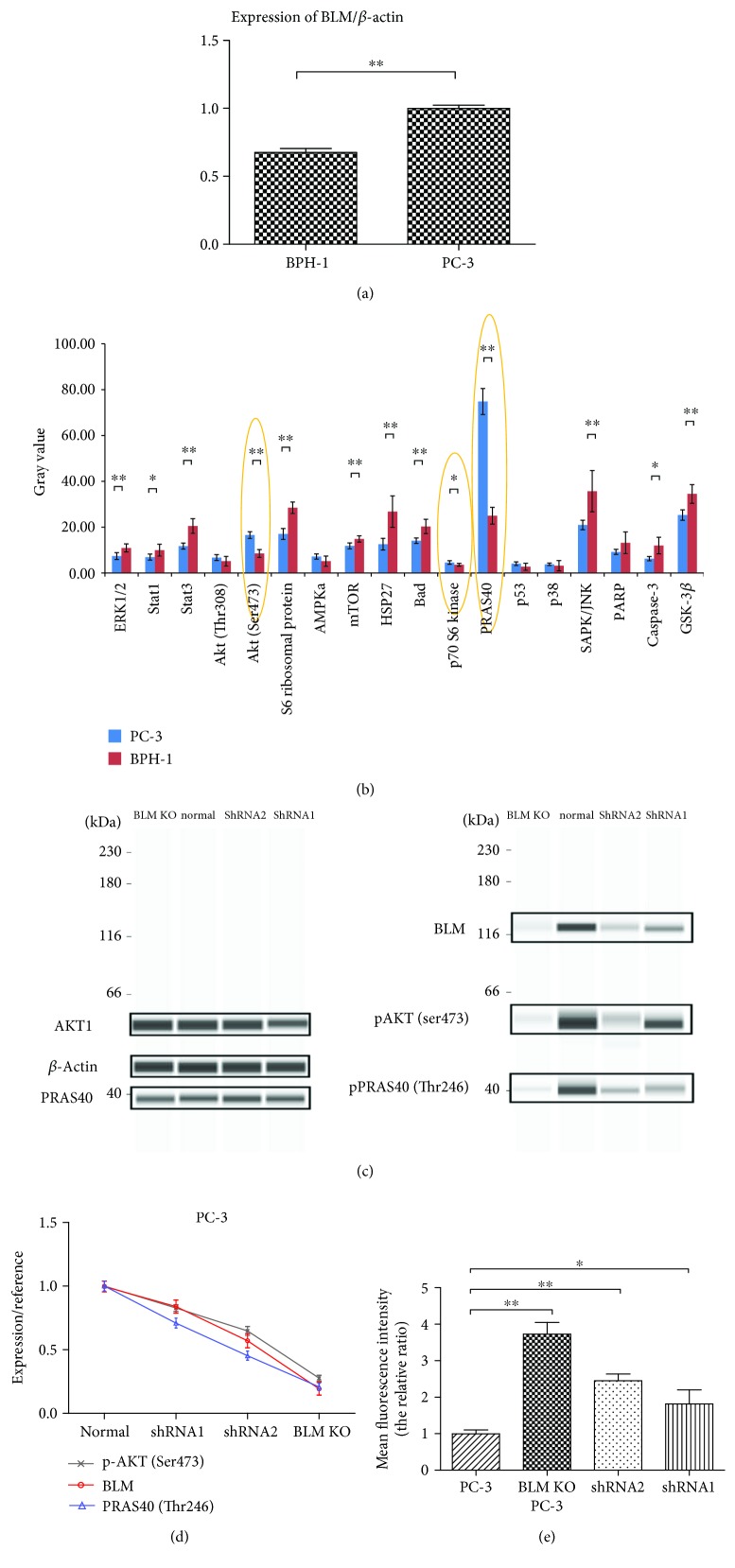
The correlation between BLM expression and phosphorylation of Akt1 and PRAS40. (a) BLM/*β*-actin expression in BPH-1 and PC-3 cells. BLM expression was lower in BPH-1 cells than in PC-3 cells. (b) Quantification of phosphorylated signaling proteins in PC-3 and BPH-1 cells. The levels of phosphorylated AKT (Ser473), p70 S6 kinase, and PRAS40 (Thr246) were lower in BPH-1 than in PC-3 cells. (c) BLM helicase, pAKT, and pPRAS40 expression in PC-3 cells was assessed by western blot and WES analysis. (d) Cells displayed significantly reduced BLM expression, as well as decreased levels of pAKT (Ser473) and pPRAS40 (Thr246). BLM expression values were normalized to *β*-actin, while pAKT and pPRAS40 (Thr246) expression values were normalized to total AKT1 and total PRAS40 expression, respectively. BLM expression was positively correlated with pAKT (*P* < 0.01) and pPRAS40 levels (*P* < 0.05). (e) Intracellular ROS levels were higher in BLM KO PC-3 cells as well as those treated with shRNA1 and shRNA2 as compared to those of PC-3 cells (mean ± SD; ^∗^ *P* < 0.05 and ^∗∗^ *P* < 0.01).

Given the effects of BLM on PC-3 cell proliferation, we next assessed whether changes in BLM expression influenced phosphorylation states within prooncogenic signaling cascades. The PathScan® Antibody Array Kit was used to analyze WT and BLM KO PC-3 cells. Previous studies have shown that BLM is more highly expressed in PC tissue than in normal prostate and benign prostatic hyperplasia tissues and that BLM regulates protein phosphorylation [40–42] (Figure 3(a)). Our screening results showed that AMPK*α*, HSP27, Bad, SAPK/JNK, and PARP expression levels were significantly upregulated in BLM KO PC-3 cells as compared to normal PC-3 cells, while AKT and PRAS40 expression levels were significantly downregulated (Figures 3(b) and 3(c)). We also observed significantly higher BLM levels in PC-3 cells than in benign prostatic hyperplasia BPH-1 cells (Figure 4(a)). When the levels of phosphorylated proliferation-associated proteins were assessed in BPH-1 cells, the levels of pAKT (Ser473), p70 S6 kinase, and pRAS40 (Thr246) were significantly downregulated (Figure 4(b)). These data confirmed that BLM influences a wide range of oncogenic signaling pathways, most notably via AKT (Ser473) and PRAS40 (Thr246) phosphorylation. In order to verify the correlation between BLM expression and the levels of phosphorylated Akt1 and PRAS40, we employed a RNAi-based approach to reduce BLM expression, revealing that levels of phosphorylated Akt1 and PRAS40 were specifically linked to BLM downregulation (Figures 4(c) and 4(d)). Given that AKT (Ser473) and PRAS40 (Thr246) phosphorylation also influence tumor growth-related ROS production, we assessed the link between BLM expression and ROS production in these cells, revealing that ROS production was significantly increased upon BLM downregulation (Figure 4(e)).

### 3.4. Effects of AKT and PRAS40 Inhibition on PC Cells

**Figure 5:**
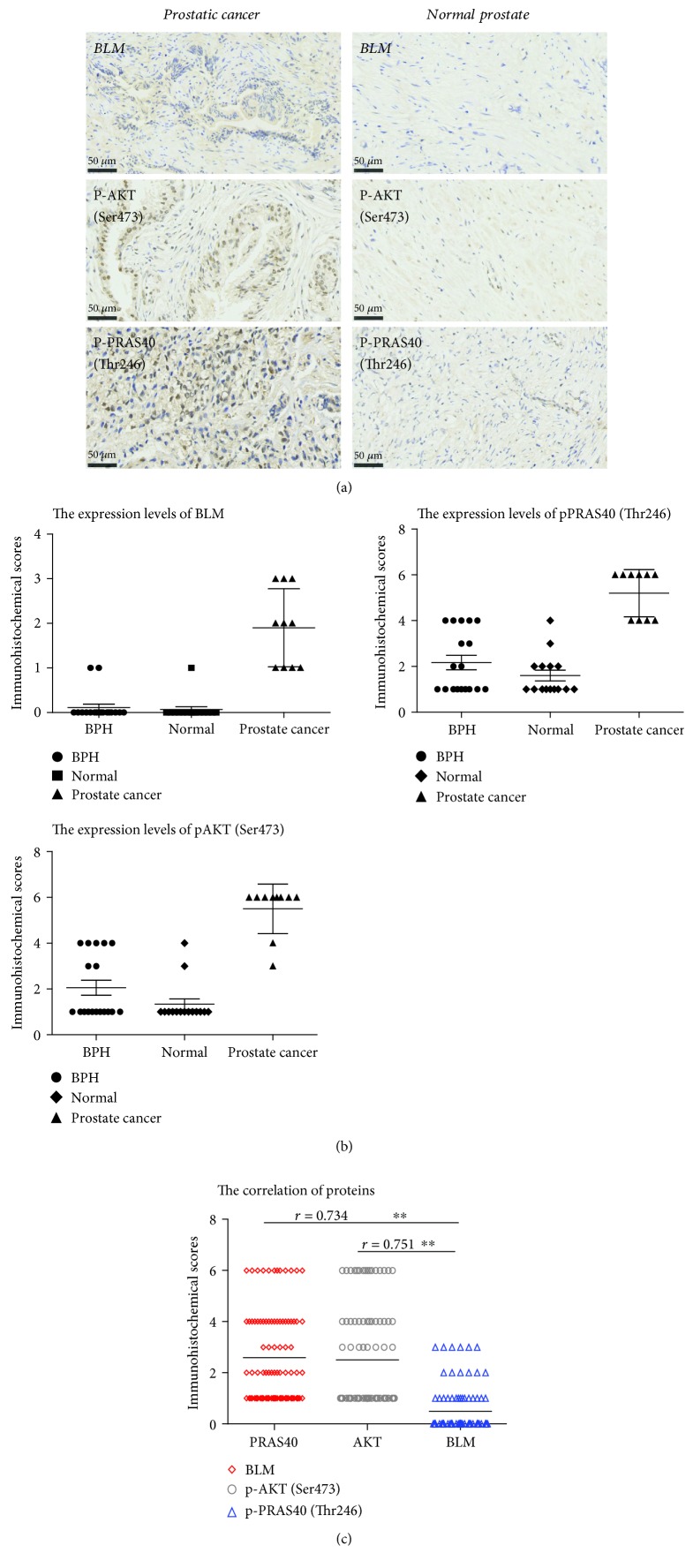
Immunohistochemical analysis of phosphoprotein levels. (a) Prostate cancer tissue: weak BLM staining intensity (10-30% positive staining), moderate pAKT (Ser473) staining (50-70% positive staining), and moderate pPRAS40 (Thr246) staining (50-70% positive staining). Normal prostate tissue: negative BLM staining (0% positive staining), moderate pAKT (Ser473) staining (10-30% positive staining), and moderate pPRAS40 (Thr246) staining (10-30% positive staining). (b) Immunohistochemical scores were calculated based on the sum of the staining intensity scores and positive staining rates. Interpretation criteria were as follows: staining intensity score: 0 (-), 1 (weak), 2 (moderate), and 3 (strong). Positive staining rates were scored as follows: 0 (negative), 1 (1-25%), 2 (26%-50%), 3 (51-75%), and 4 (76%-100%). pAKT (Ser473), pPRAS40 (Thr246), and BLM expression in prostate cancer tissue was higher than in other tissues. (c) BLM expression positively correlated with pAKT (*r* = 0.751, *P* < 0.01) and pPRAS40 (Thr246) expression (*r* = 0.751, *P* < 0.01).

**Figure 6:**
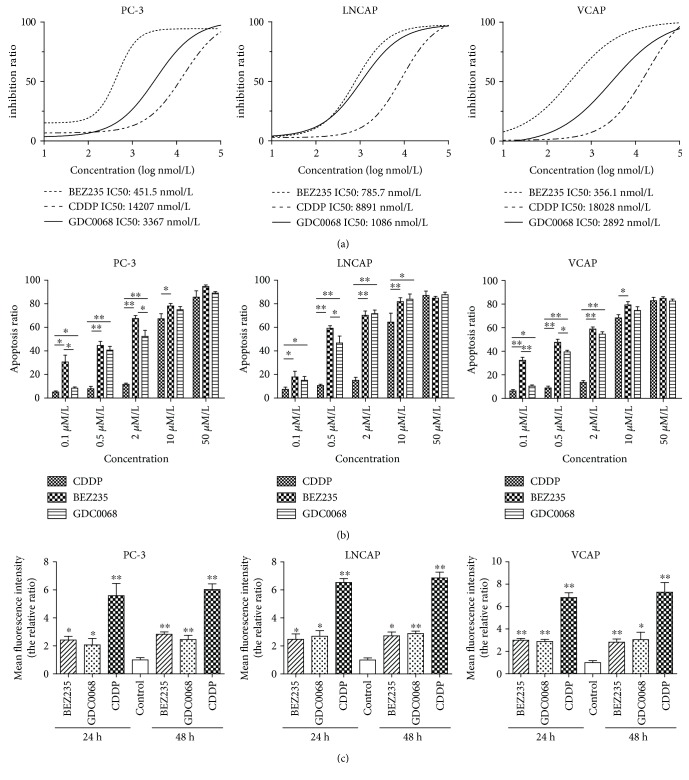
BEZ235 and GDC-0068 reduce proliferation and induce apoptosis more potently than CDDP in PC cells. (a) Inhibition ratios in prostate cancer cells following 48 h treatment: for PC-3 cells, BEZ235 IC50—451.5 ± 79.23 nmol/L, CDDP IC50—14207 ± 96.49 nmol/L, and GDC-0068 IC50—3367 ± 97.79 nmol/L; for LnCAP cells, BEZ235 IC50—785.7 ± 94.4 nmol/L, CDDP IC50—8891 ± 100.7 nmol/L, and GDC-0068 IC50—1086 ± 94.7 nmol/L; and for VCAP cells, BEZ235 IC50—356.1 ± 99.07 nmol/L, CDDP IC50—18028 ± 113.9 nmol/L, and GDC-0068 IC50—2892 ± 105.8 nmol/L (*P* < 0.05). (b) Apoptosis ratios in various prostate cancer cell lines after drug treatments. At a concentration of 0.5 *μ*mol/L, BEZ235 and GDC-0068 induced apoptosis in ~40% of cancer cells. CDDP induced lower relative levels of apoptosis (~20%; 24 h treatment). (c) Cells were treated with BEZ235 (2 *μ*mol/L), GDC-0068 (2 *μ*mol/L), and CDDP (10 *μ*mol/L) for 24 and 48 hours. GDC-0068, BEZ235, and CDDP significantly increased ROS production in tumor cells. Values represent the mean ± standard deviation (SD); ^∗^ *P* < 0.05 and ^∗∗^ *P* < 0.01.

We found that phosphorylated AKT and PRAS40 levels were lower when BLM expression was reduced in PC cell lines. We performed immunohistochemical staining to validate these finding in PC tissue samples. According to the cytoplasmic staining intensity and positive staining rates, pAKT (Ser473), pPRAS40 (Thr246), and BLM expression levels were higher in prostate cancer tissue than in non-PC tissues (Figure 5). To investigate the effects of AKT and PRAS40 activity in PC cell lines, PC-3, LnCAP, and VCAP cells were treated with BEZ235, a highly specific AKT inhibitor [43–45], and GDC-0068, a PRAS40 inhibitor [46, 47]. Cell viability was then assessed via a CCK-8 assay. Cisplatin (CDDP) was included in these experiments as a positive control. All three drugs reduced the proliferation of all the PC cell lines, with BEZ235 having the lowest half maximal inhibitory concentration (IC50), followed by that of GDC-0068 and that of CDDP (Figure 6(a)). Annexin V-FITC/PI staining was used in conjunction with flow cytometry to investigate whether BEZ235, GDC-0068, and CDDP induced apoptosis in these PC cell lines following 24 h of treatment. Relatively low concentrations of BEZ235 and GDC-0068 induced significant levels of apoptosis compared to CDDP (Figure 6(b)), confirming that they exert a strong apoptotic effect in PC cancer cells. Cisplatin is known to mediate ROS production in PC cells [48], and it is possible that similar mechanisms contributed to the observed induction of apoptosis mediated by these additional inhibitory compounds (Figure 6(c)).

### 3.5. Impact of AKT (Ser473) and PRAS40 (Thr246) Phosphorylation on BLM Expression

**Figure 7:**
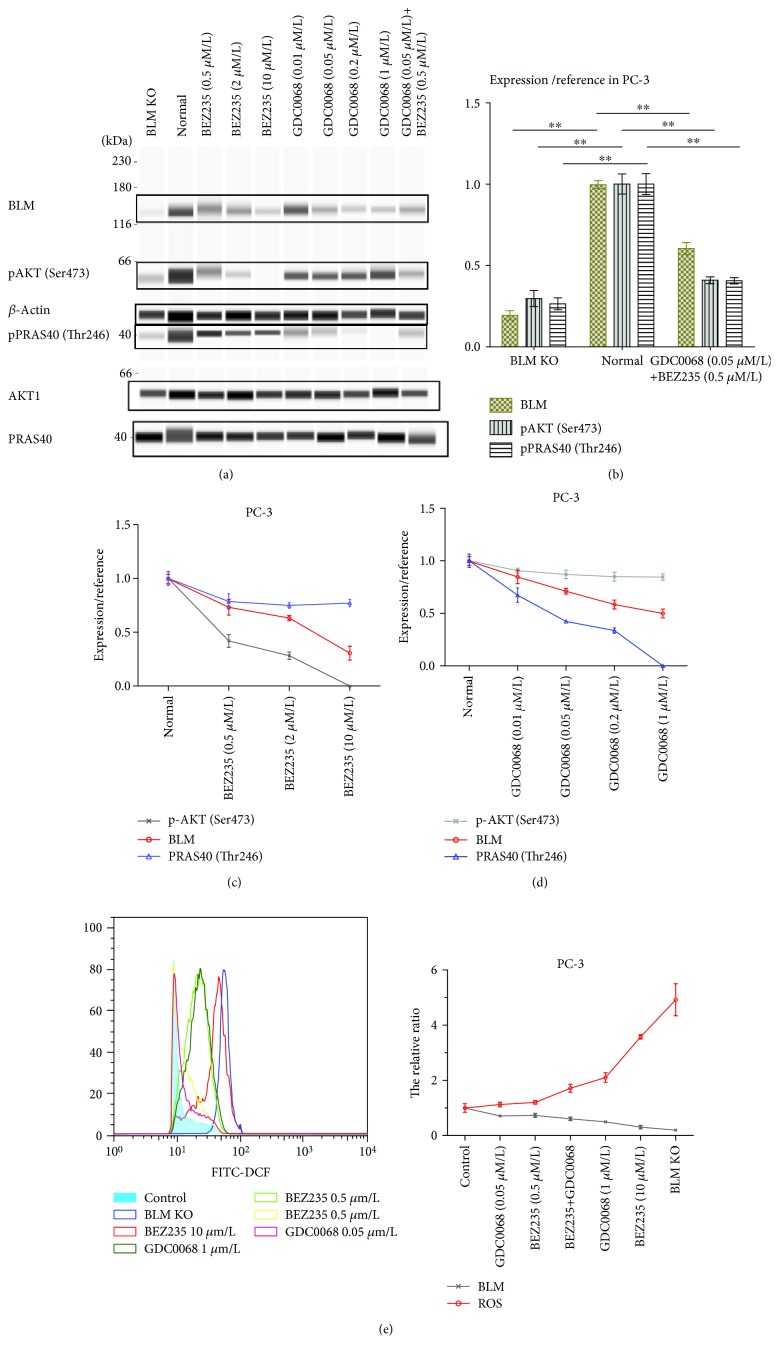
BLM, pAKT, and pPRAS40 expression in PC-3 cells. (a) BLM helicase, pAKT, and pPRAS40 expression in PC-3 cells as measured by western blot and WES analysis. (b) Cells treated with BEZ235 and GDC-0068 displayed significantly reduced BLM expression, while BLM knockout decreased pAKT (Ser473) and pPRAS40 (Thr246) levels. BLM expression values were normalized to *β*-actin, and pAKT and pPRAS40 (Thr246) expression values were normalized to total AKT1 and PRAS40, respectively. Data represent the mean ± SD (^∗^ *P* < 0.05 and ^∗∗^ *P* < 0.01). BLM expression is positively correlated with (c) pAKT (*P* < 0.05) and (d) pPRAS40 levels (*P* < 0.01). (e) ROS production is negatively correlated with BLM expression (*P* < 0.05).

**Figure 8:**
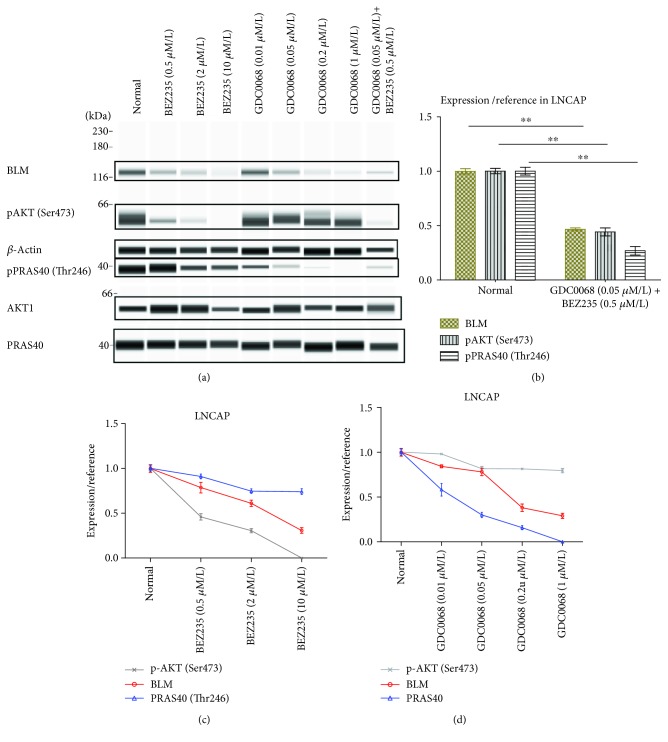
BLM, pAKT, and pPRAS40 expression in LNCAP cells. (a) BLM helicase, pAKT, and pPRAS40 expression in LNCAP cells as measured by western blot and WES analysis. (b) Cells treated with BEZ235 and GDC-0068 displayed significantly reduced BLM expression. BLM expression values were normalized to *β*-actin, and pAKT and pPRAS40 (Thr246) expression values were normalized to total AKT1 and PRAS40, respectively. Data represent the mean ± SD (^∗^ *P* < 0.05 and ^∗∗^ *P* < 0.01). BLM expression is positively correlated with (c) pAKT (*P* < 0.05) and (d) pPRAS40 levels (*P* < 0.05).

Our results so far have shown that BLM is required for AKT and PRAS40 phosphorylation. Given that BLM, pAKT, and pPRAS40 all displayed reduced expression in non-PC tissue, we assessed whether inhibiting AKT and PRAS40 could also influence BLM expression. For these experiments, PC-3 and LnCAP cells were treated with BEZ235 and GDC-0068 followed by assessment of BLM, pAKT, and pPRAS40 levels using WES (Figures 7 and 8). Treating cells with BEZ235 and GDC-0068 resulted in a significant decrease in BLM expression. Drug activity was confirmed by measuring downregulation of pAKT and pPRAS40 following their respective drug treatments. These findings suggested that BLM expression positively correlates with AKT and PRAS40 activity and that inhibiting these kinases downregulates BLM expression in PC cells. Increases and decreases in ROS serve as a double-edged sword, and both can mediate tumor occurrence and development. Therefore, it is feasible that altered BLM expression may contribute to this process in a similar way. We found that reductions in BLM expression in PC-3 cells were associated with increased ROS production and that these increases occurred in a dose-dependent manner (Figure 7(e)).

## 4. Discussion

As a result of the aging population and lifestyle changes, the incidence of PC continues to increase worldwide, lending an urgency to the requirement for new and effective PC treatments. Aging is a key mediator of cancer development, both for probabilistic reasons and potentially due to increased ROS production which is linked with both cancer and with so-called “inflamm-aging” processes [49]. In this study, through an iTRAQ-mediated analysis of PC clinical samples, we screened for differentially expressed proteins in human PC tissue, normal prostate tissue, and benign prostatic hyperplasia tissue in order to identify DEPs which may represent novel anti-PC targets. Our results showed that BLM expression was significantly increased in PC tissue, and the results of a GO term enrichment analysis revealed that BLM regulates protein phosphorylation in PC cells (GO: 0001932). We further determined that BLM downregulation reduced both pAKT and pPRAS40 levels, suggesting that their activity is dependent upon BLM expression. Importantly, we found that BLM downregulation, AKT inhibition, and PSRAS40 inhibition all resulted in enhanced ROS production, reduced PC cell proliferation, and increased apoptosis. This highlights AKT and PSRAS40 signaling as potential mediators of BLM-induced PC tumorigenesis, suggesting that this signaling axis could be a novel target for PC therapy.

BLM is a helicase implicated in homologous recombination, a key pathway involved in the repair of double-strand DNA breaks [40]. Helicases unwind DNA or RNA and are important for maintaining genome integrity during DNA replication and repair. Therapies that target DNA damage repair enzymes have recently shown promise as a chemotherapeutic strategy due to cancer-associated genomic instability [50, 51]. This led to the characterization of the first BLM helicase inhibitor (termed ML216); however, this compound lacked specificity and also inhibited the ability of closely related helicases to unwind DNA, leading to off-target effects [52, 53]. In this study, GO analysis annotated BLM expression in PC cells as a “regulatory process for protein phosphorylation,” as BLM participates in the regulation of cyclin-dependent protein serine/threonine kinase activity. BLM expression is lowest in the G1 phase, significantly increases in the S phase, and peaks in the G2/M phase of the cell cycle [54, 55]. BLM binds to the spindle assembly checkpoint kinase, monopolar spindle 1 (MPS1), and is phosphorylated at Ser144. Phosphorylated BLM may then bind to Polo-like kinase 1 (PLK1) via its polo-box domain (PBD), further contributing to the maintenance of genomic stability in both healthy [56, 57] and tumor tissues [58–61]. Together, these data highlight the multifunctional roles of BLM and its ability to regulate multiple different oncogenic pathways.

Precision cancer medicine has emerged as a modern cancer treatment strategy and has enabled more accurate and efficient therapeutic treatment for individual cancer patients. Precision medicine employs tumor genomic profiling to inform treatment decisions [62–67]. This is the result of identifying numerous cancer drivers, alterations in their activity, and therapies that target these alterations. PI3K is the major activator of AKT, and this PI3K signaling pathway is commonly mutated in PC, often through the downregulation of PTEN, PIK3CA/B amplification, or activating mutations of PIK3CA/B and AKT1 [68]. Interestingly, AKT was also identified in the present study. Indeed up to 49% of PC tumors are thought to have aberrant PI3K signaling, making it the 2nd most frequently altered pathway in PC [68–70]. However, PI3K monotherapies for PC are ineffective, most likely due to their lack of specificity and complex signaling feedback mechanisms [68, 69, 71–74]. Recently, clinical trials have been initiated for inhibitors of specific PI3K isoforms and may provide increased specificity. In addition, the combination of Ipatasertib and Abiraterone in PTEN-null prostate cancer has improved PC patient survival in phase II studies, demonstrating the efficacy of the reciprocal targeting of PTEN loss and PI3K [75].

In this study, we identified a novel reciprocal relationship in PC tissue between BLM-AKT and PRAS40 and demonstrated that BLM, AKT, and PSRAS40 inhibition was beneficial for PC treatment *in vitro*. Using CRISPR/Cas9-mediated homologous recombination to perform site-directed knockout of the BLM gene in PC-3 cells, we demonstrated that BLM KO leads to a reduction in PC cell proliferation. Similarly, prior studies have shown that BLM silencing using RNAi interference inhibited the proliferation, migration, and invasion of PC-3 cells [40]. Interestingly, PLK1 phosphorylates and inactivates PTEN and is regulated in part by BLM. BLM is also involved in the regulation of the G2/M cell cycle checkpoint and mitotic progression, and BLM expression levels fluctuate with the cell cycle and peak during the G2/M phase. In addition, phosphorylation of PRAS40 can activate 14-3-3, and the 14-3-3 protein can promote PLK1 catalytic activity to control mitotic progression and the G2/M checkpoint [76]. BLM may therefore use PLK1 as an intermediary to affect AKT (Ser473) and PRAS40 (Thr246) phosphorylation. In addition, since BLM expression is related to the cell cycle, increased BLM expression indicates that an increased proportion of cells are in the G2/M phase. Given the intense research into the discovery of novel potent and selective BLM inhibitors with improved physiochemical properties [52, 53, 77], future efforts to utilize BLM and AKT inhibitors in a combination therapy to treat PC tumors may represent a viable target for precision medicine.

While our primary focus in this study was to observe the differences in the regulation of BLM-mediated protein phosphorylation, ROS levels are known to be key players in oncogenic processes, with marked elevations in ROS levels in many tumor systems indicating the presence of a stressful redox environment in these cells [78]. Some of the DEPs identified in the present study are linked to ROS-related pathways in addition to their association with the regulation of protein phosphorylation. For example, ROS produced in response to the activation of growth factor signaling pathways can mediate the activation of key kinases in the mitogen-activated protein kinase (MAPK), extracellular signal-regulated kinase (ERK), and focal adhesion kinase families, thereby enhancing the migration and invasive potential of cancer cells [79]. Importantly, ROS have been shown to modulate signaling in order to promote AKT upregulation and PTEN downregulation both *in vitro* and *in vivo* [80].

We further confirmed that ROS production is negatively correlated with BLM expression. Multiple studies have shown that ROS-mediated damage can cause substantial disruptions in cellular protein function and DNA integrity. This is one of the reasons underlying the susceptibility of BS patients with BLM mutations to multiple malignancies. In addition, BLM expression positively correlates with AKT and PRAS40 activity in PC cells. Enhancing PI3K/AKT/mTOR pathway signaling plays a critical role in determining the lifespan and cellular senescence of mammalian cells. Since AKT and RAS40 were downregulated, this may be one of the reasons why BS patients do not exhibit a progeria phenotype. BLM, pAKT, and pPRAS40 all display increased expression in PC tissues, and ROS is similarly increased in this context [81]. ROS and BLM may cooperate with one another in order to promote tumorigenesis. As a promoter of DNA replication, the high BLM expression satisfies the high replicative demands of cancer cells, while the ability of BLM to maintain genomic stability confers survival advantages to these malignant cells. ROS production is associated with increased metabolic rates and DNA damage. Thus, the correlation between BLM and ROS production suggests that BLM is involved in repairing this ROS-mediated DNA damage.

## 5. Conclusions

Our results have revealed that BLM overexpression and subsequent changes in protein phosphorylation contribute to PC progression. BLM, pAKT, and pPRAS40 levels are significantly increased in PC tissue, and the expression levels of these proteins are closely related, with BLM inhibition leading to a loss of pAKT/PRAS40 and vice versa. While a large number of studies have implicated AKT and PRAS40 hyperactivity in promoting tumor cell proliferation, the correlation between these signaling molecules and BLM has not been previously reported. In addition, we have demonstrated that BLM, AKT, and PSRAS40 influence the proliferative capacity of PC cells and that their inhibition enhances ROS production and leads to PC-3 cell apoptosis. These novel results suggest that ROS-related mechanisms may contribute to PC pathogenesis. Combining BLM and AKT inhibitors with therapeutic modalities targeting ROS generation may facilitate the achievement of durable responses against PC as a consequence of these effective multimodal approaches.

## Figures and Tables

**Table 1:** DEPs involved in the regulation of protein phosphorylation (GO: 0001932).

Accession	UniProtKB	Gene	Cancer/normal	Cancer/normal	Cancer/BPH	Cancer/BPH
Number	AC	Name	118, 119, 121/113, 114	*P* value	118, 119, 121/115, 116, 117	*P* value
*Upregulated proteins* ^a^						
Q9Y4K3	Q9Y4K3	TRAF6	1.470	0.002	1.246	0.004
Q96B36	Q96B36	AKT1S1	1.212	0.006	1.218	0.001
Q8TB45	Q8TB45	DEPTOR	1.446	0.002	1.221	0.002
Q59FU8	P25445	FAS	1.606	0.013	1.239	0.020
Q02750	Q02750	MAP2K1	1.225	0.004	1.227	0.000
P54132	P54132	BLM	1.258	0.001	1.215	0.001
P31749	P31749	AKT1	1.207	0.010	1.205	0.001
*Downregulated proteins* ^b^						
A0A1V1FWL6	P49815	TSC2	0.706	0.001	0.786	0.002
A0A024R593	Q15173	PPP2R5B	0.719	0.002	0.777	0.004

^a^Proteins with fold changes > 1.2 (*P* < 0.05) are considered upregulated. ^b^Proteins with fold changes < 0.8 (*P* < 0.05) are considered downregulated.

**Table 2:** Target pathways identified by KEGG enrichment.

Map name	Map ID	Input number	Input proteins	*P* value
Pathways in cancer	K05200	8	Q02750|P31749|P50150|P25445|P06753|Q9Y4K3|Q15147|P11166	1.45*E* − 08
Thyroid hormone signaling pathway	K04919	5	P11166|Q02750|P31749|Q15147|P49815	3.03*E* − 07
mTOR signaling pathway	K04150	5	Q02750|Q8TB45|P49815|P31749|Q96B36	7.88*E* − 07
Adrenergic signaling in cardiomyocytes	K04261	5	Q15173|P31749|P06753|Q9BXT2|Q15147	1.04*E* − 06
p53 signaling pathway	K04115	4	P25445|P27701|P31350|P49815	1.32*E* − 06
PI3K-Akt signaling pathway	K04151	6	Q02750|P31749|P50150|P22105|Q15173|P49815	2.55*E* − 06
Chagas disease (American trypanosomiasis)	K05142	4	P25445|P31749|Q9Y4K3|Q15147	1.06*E* − 05
MAPK signaling pathway	K04010	5	P31749|P25445|Q02750|Q9BXT2|Q9Y4K3	1.09*E* − 05
Cholinergic synapse	K04725	4	Q02750|P31749|P50150|Q15147	1.32*E* − 05
Sphingolipid signaling pathway	K04071	4	Q15173|Q02750|P31749|Q15147	1.44*E* − 05
AMPK signaling pathway	K04152	4	Q15173|P49815|P31749|Q96B36	1.59*E* − 05
Dopaminergic synapse	K04728	4	Q15173|P31749|P50150|Q15147	2.26*E* − 05
Phospholipase D signaling pathway	K04072	4	Q02750|P31749|Q15147|P49815	2.91*E* − 05
Chemokine signaling pathway	K04062	4	Q02750|P31749|P50150|Q15147	7.69*E* − 05

*P* value < 0.05, the number of target proteins involved in the pathway ≥4.

## Data Availability

Datasets used in the study are available from the corresponding author upon reasonable request.

## Abbreviations

BLM: Bloom syndrome protein
WES: Automated western blot analysis system
iTRAQ: Isobaric tag for relative and absolute quantitation
TRAF6: TNF receptor-associated factor 6
AKT1S1: AKT serine/threonine kinase 1 substrate 1
DEPTOR: DEP domain-containing MTOR interacting protein
MAP2K1: Mitogen-activated protein kinase 1
TSC2: Tuberous sclerosis complex subunit 2
PPP2R5B: Serine/threonine-protein phosphatase 2A 56 kDa regulatory subunit beta isoform
PLK1: Polo-like kinase 1
14-3-3: Tyrosine 3-monooxygenase/tryptophan 5-monooxygenase activation protein theta
ROS: Reactive oxygen species.

## Supplementary Materials

Supplementary Materials Figure 1: PC-3 cells were identified by STR authentication. Figure 2: it is proven by sequence analysis that sgRNA are correctly inserted into the expression vector. Figure 3: the construction of BLM helicase CRISPR/Cas9 donor vector. Figure 4: the target efficiency was analyzed by T7E1 enzyme digestion. Figure 5: expression of BLM helicase gene mRNA in PC-3 cells. Table1: the DEP list among prostate cancer, normal prostate, and benign prostatic hyperplasia tissues was identified using iTRAQ.
