# Supplementary material for: Bloom Syndrome Protein Activates AKT and PRAS40 in Prostate Cancer Cells
Source: Oxid Med Cell Longev. 2019 May 9;2019:3685817. doi: 10.1155/2019/3685817 (PMC6532288; doi:10.1155/2019/3685817)
Supplement: Supplementary Materials — Figure 1: PC-3 cells were identified by STR authentication. Figure 2: it is proven by sequence analysis that sgRNA are correctly inserted into the expression vector. Figure 3: the construction of BLM helicase CRISPR/Cas9 donor vector. Figure 4: the target efficiency was analyzed by T7E1 enzyme digestion. Figure 5: expression of BLM helicase gene mRNA in PC-3 cells. Table1: the DEP list among prostate cancer, normal prostate, and benign prostatic hyperplasia tissues was identified using iTRAQ. [file 3685817.f1.pdf]

10 Loci STR Profile:

Genetic Site	ATCC		Customer sample	
(Locus)	PC-3		PC-3	
Amelogenin	X		X	
CSF1PO	11		11	
D13S317	11		11	
D16S539	11		11	
D5S818	13		13	
D7S820	8	11	8	
THO1	6	7	6	7
TPOX	8	9	8	9
vWA	17		17	
D21S11			29	31.2
Percent match between the sample and the database profile: 95.7%				

Figure1. PC-3 cells were identified by STR authentication.

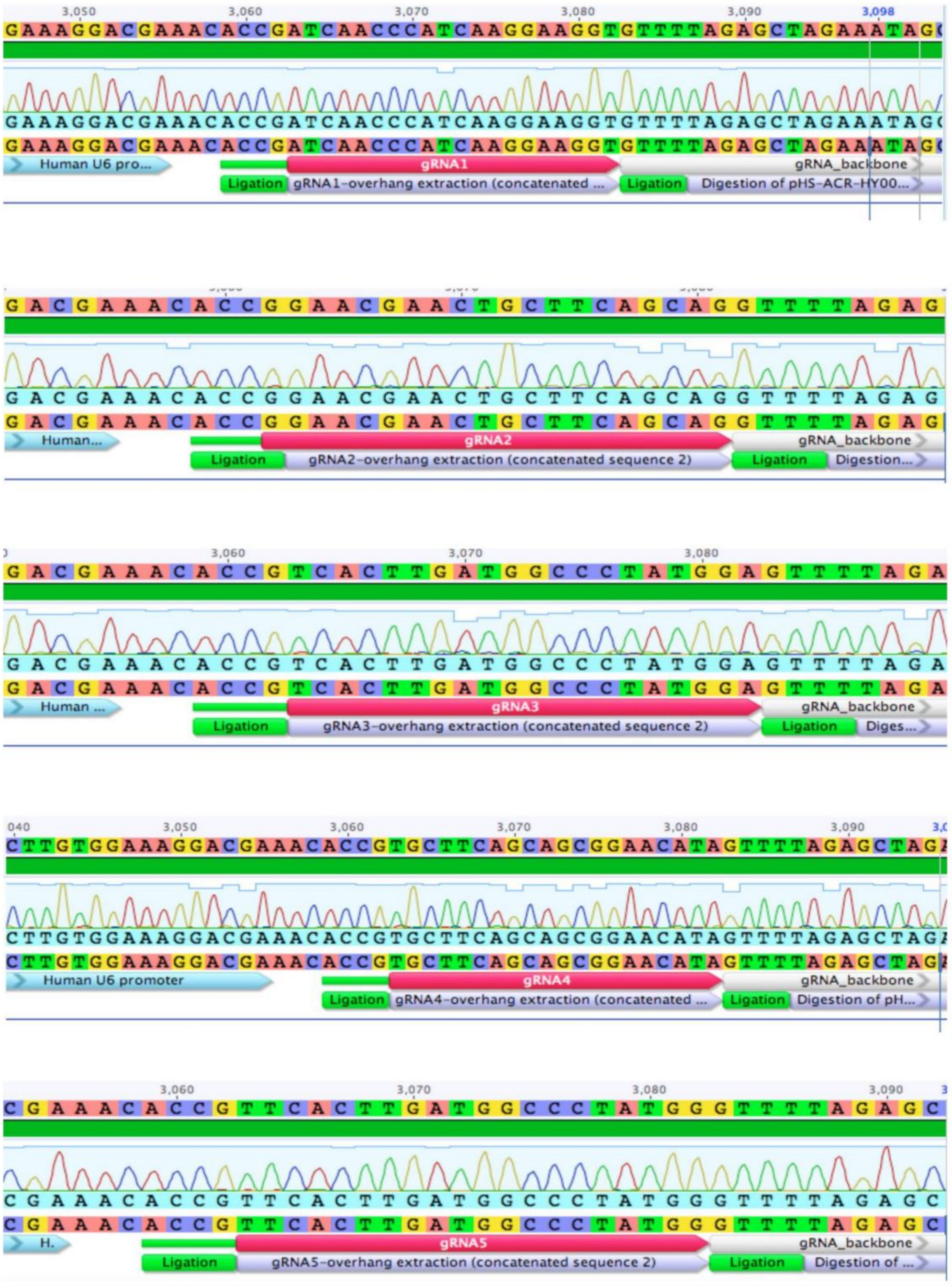

Figure2. It is proved by sequence analysis that sgRNA are correctly inserted into the expression vector.

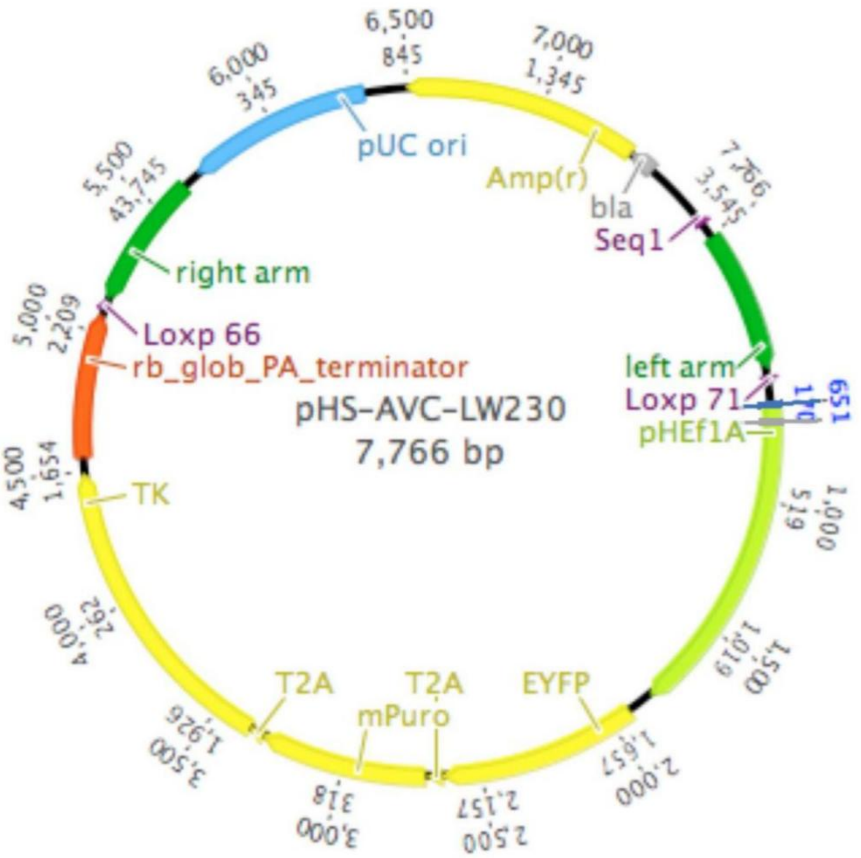

Figure3. The construction of BLM helicase CRISPR/Cas9 donor vector.

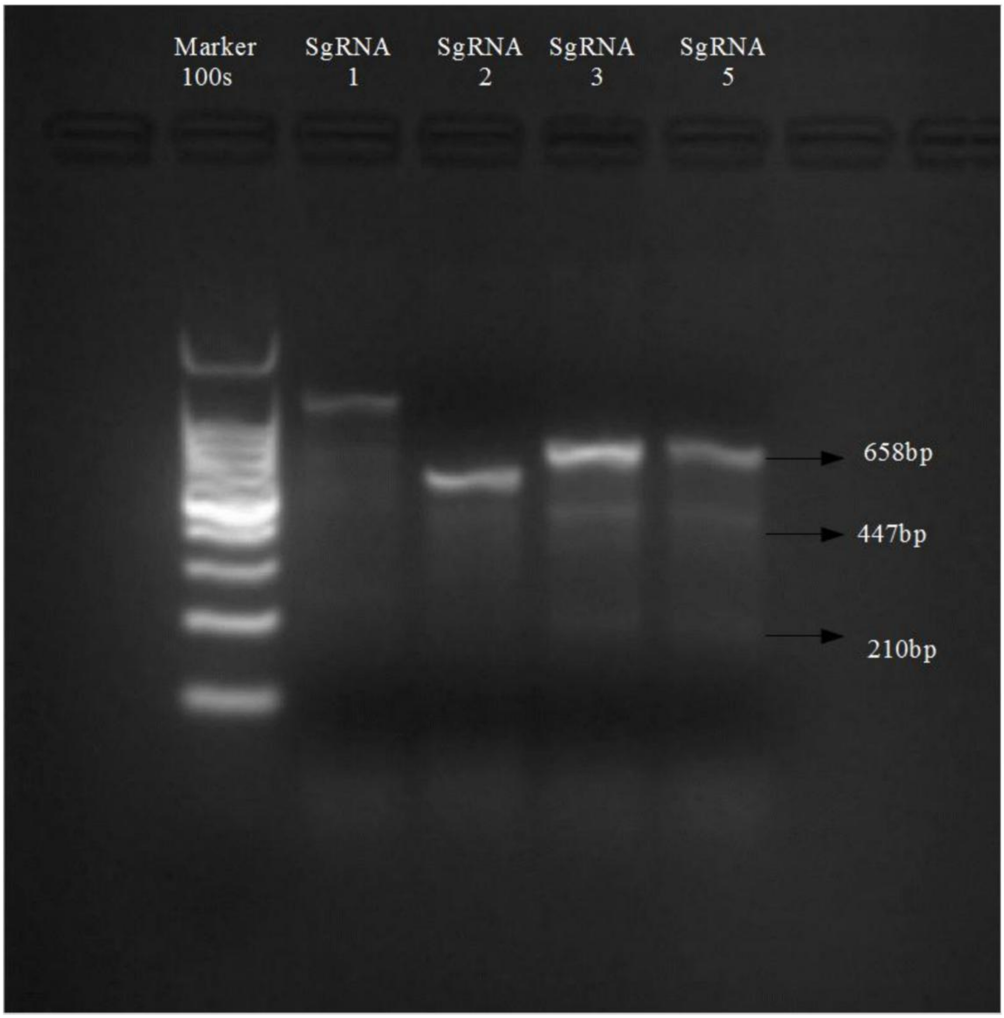

Figure4.The target efficiency was analyzed by T7E1 enzyme digestion

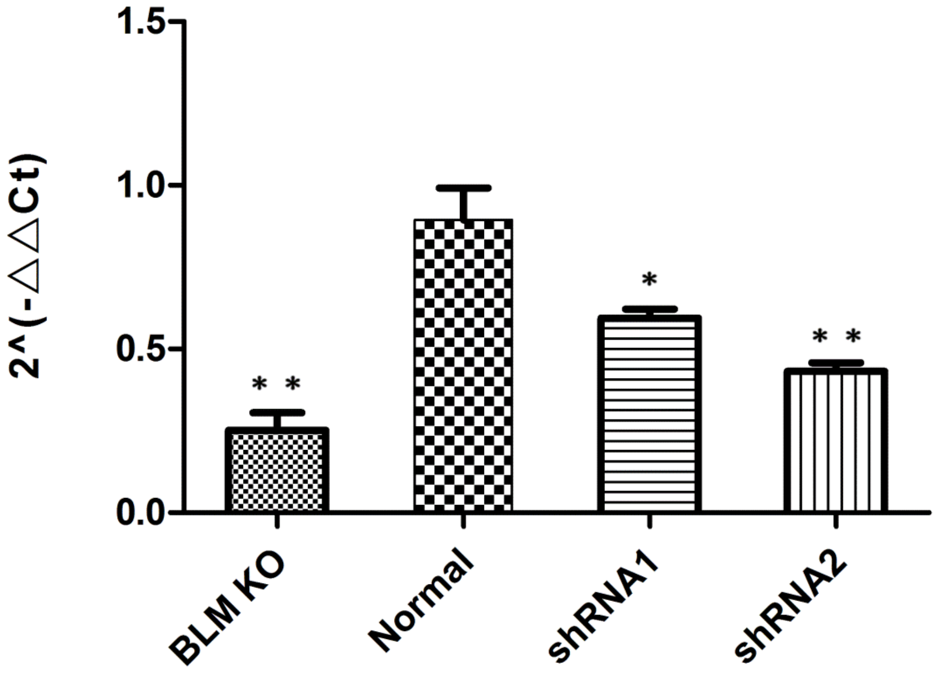

Figure5.Expression of BLM helicase gene mRNA in PC-3 cells.

Table1. The DEPs list amongst prostate cancer, normal prostate, and benign prostatic hyperplasia tissues were identified using iTRAQ

Accession Number	UniProtKB AC	Gene Name	Cancer/Normal 118, 119, 121/113, 114	Cancer/Normal P-value	Cancer/BPH 118, 119, 121/115, 116, 117	Cancer/BPH P-value
up-regulated proteins[a]						
Q9Y4K3	Q9Y4K3	TRAF6	1.470	0.002	1.246	0.004
Q9BXT2	Q9BXT2	CACNG6	1.317	0.004	1.217	0.000
Q96B36	Q96B36	AKT1S1	1.212	0.006	1.218	0.001
Q8TB45	Q8TB45	DEPTOR	1.446	0.002	1.221	0.002
Q59GY2	P11166	SLC2A1	1.216	0.007	1.216	0.001
Q59FU8	P25445	FAS	1.606	0.013	1.239	0.020
Q15147	Q15147	PLCB4	1.206	0.033	1.228	0.003
Q04656	Q04656	ATP7A	1.462	0.002	1.212	0.012
Q02750	Q02750	MAP2K1	1.225	0.004	1.227	0.000
P54132	P54132	BLM	1.258	0.001	1.215	0.001
P50150	P50150	GNG4	1.404	0.000	1.223	0.003
P31749	P31749	AKT1	1.207	0.010	1.205	0.001
D6W4Z6	P31350	RRM2	1.735	0.003	1.457	0.001
B3KY94	O14735	CDIPT	1.350	0.009	1.221	0.010
A0A0S2Z4G8	P06753	TPM3	2.321	0.002	1.217	0.012
A0A0A0N0N2	P26992	CNTFR	1.588	0.006	1.292	0.008
A0A087WWA5	P22105	TNXB	1.336	0.004	1.200	0.001
Down-regulated proteins[b]						
P48426	P48426	PIP4K2A	0.666	0.002	0.799	0.005
P27701	P27701	CD82	0.694	0.001	0.774	0.000
A0A1V1FWL6	P49815	TSC2	0.706	0.001	0.786	0.002
A0A024R593	Q15173	PPP2R5B	0.719	0.002	0.777	0.004

Note:

[a] Proteins with fold changes＞1.2 (P＜0.05) are considered to be up-regulated.

[b] Proteins with fold changes＜0.8 (P＜0.05) are considered to be down-regulated.
